# Development of an isothermoal amplification-based assay for rapid visual detection of an Orf virus

**DOI:** 10.1186/s12985-016-0502-x

**Published:** 2016-03-19

**Authors:** Yang Yang, Xiaodong Qin, Guangxiang Wang, Jiaxin Jin, Youjun Shang, Zhidong Zhang

**Affiliations:** State Key Laboratory of Veterinary Etiological Biology, Key Laboratory of Grazing Animal Diseases of Ministry of Agriculture, Lanzhou Veterinary Research Institute, Chinese Academy of Agriculture Sciences, Lanzhou, Gansu China; School of Life Science, Lanzhou University, Lanzhou, Gansu China

**Keywords:** Orf virus, Recombinase polymerase amplification, RPA, Lateral flow, Rapid test, Isothermal nucleic acid amplification

## Abstract

**Background:**

Orf virus (ORFV) is the causative agent of a severe infectious skin disease (also known as contagious ecthyma) in goats, sheep and other small ruminants. Importantly, ORFV also infect humans which causes a public health concern in the context of changing environment and increase in human populations. The rapid detection is critical in effective control of the disease and urgently needed.

**Results:**

A novel “point of care” molecular amplification assay for rapid visual detection of ORFV was developed based on isothermoal recombinase polymerase amplification (RPA) technology in combination with a simpler lateral flow immunoassay strip (ORFV RPA- LFD assay). The developed ORFV RPA- LFD assay was able to detect ORFV in less than 25 min. This assay was highly sensitive, with detection limit of as low as 80 copies/reaction, and highly specific, with no cross-reactions with capripox virus, foot-and-mouth disease virus and peste des petits ruminants virus. Furthermore, the ORFV RPA- LFD assay has good correlation with qPCR assay for detection of ORFV present in clinical samples.

**Conclusions:**

The developed ORFV RPA-LFD assay was a sensitive and specific method for rapid detection of ORFV, and has great potential as an onsite molecular diagnostic tool in control of Orf.

**Electronic supplementary material:**

The online version of this article (doi:10.1186/s12985-016-0502-x) contains supplementary material, which is available to authorized users.

## Background

Orf, also known as contagious ecthyma, is a skin disease caused by Orf virus (ORFV), which is classified in the genus Parapoxvirus of the family Poxviridae. Orf principally affects goats and sheep with worldwide distribution and significant financial importance. The clinical symptoms of Orf are characterized by the formation of papules, vesicles and growing scabs on the lips and muzzle of infected animals. ORFV is also a zoonotic parapoxvirus endemic to most countries in the world and is principally associated with small ruminants [1]. Humans of infection with ORFV is thought to be under diagnosed and causes a public health concern in the context of changing environment and increase in human populations because it can resemble skin lesions associated with potentially life-threatening zoonotic infections such as tularemia, cutaneous anthrax, and erysipeloid. Therefore, rapid and definitive diagnosis is critical and highly needed for confirmation and epidemiological investigations [1].

At the present, molecular amplification assays, in particular real-time quantitative PCR (qPCR) and loop-mediated isothermal amplification (LAMP) are sensitive for detection of ORFV [2–5]. However, due to the high complexity of assays or dependency of the specialized and rather expensive equipments, none of these tests can be easily adapted to be a Point of care test or are widely used in resource-limited settings where the disease is mostly endemic [4, 6, 7]. In recent years, a novel and rapid isothermal molecular diagnostic approach, termed recombinase polymerase amplification(RPA), has been developed as an alternative to PCR assay and LAMP [8]. In this assay, just two target specific oligonucleotide primers are used to bind the template DNA with the assistance of a recombinase in combination with strand-displacement DNA synthesis. Importantly, an amplification of DNA targets can be achieved in less than 30 min at temperatures just above room temperature. Since its initial development in 2006, a different detection format of RPA assays including probe based and lateral flow dipstick (LFD) detection has been successfully developed for rapid detection of various pathogens [8–22]. To meet our need for rapid assay on side and resource-limited settings, a RPA assay in combination with a simpler LFD is a desirable option. Most recently, we successfully developed fluorescent probe-based RPA assay for detection of ORFV with high sensitivity and specificity [23]. In the present study, a PRA assay in combination with a simpler LFD (designated as ORFV RPA-LFD assay) has been developed and evaluated for rapid detection of ORFV. To the best of our knowledge, it is for the first time a rapid molecular amplification assay was developed for on site detection of ORFV. After determination of the sensitivity and specificity of the assay, clinical samples from sheep were tested and compared with results from the corresponding qPCR assay.

## Methods

### Virus and cells

All viruses used in this study were preserved in our laboratory: ORFV/Vaccine/CHA, ORFV/Gansu/CHA, ORFV/HB/CHA; Capripox virus CHA vaccine strain, Capripox virus/Henan/CHA; peste des petits ruminants virus (PPRV) Nigeria 75/1; foot-and-mouth disease virus (FMDV)/O/CHA, FMDV/A/CHA and FMDV/Asia 1/CHA.

### Sample preparation

Twenty four field samples (*n* = 24) were collected from goats with suspected orfv infection and eight nasal swabs(swab up the nostrils of the animals concerned) collected from eight experimentally infected sheep as previously described [23]. To prepare ORFV-spiked tissues lysates, ORFV-free tissues samples of skin, lymphatic nodes, liver, lungs, stomach and kidney (*n* = 24, three each tissue) were collected from four healthy sheep. ORFV is an epitheliotropic virus, therefore sample of skin tissue was selected. In addition, samples of other tissues were also chosen because various types of tissues are often received in the field diagnostics for differential diagnosis, therefore, it would be critical to access the compatibility of the developed assay with different tissues matrix. 10 % (w/vol) tissue suspensions were prepared as previously described [23], and then tissue lysates were spiked with 10^4^ TCID_50_ of ORFV/HB/CHA and stored at -80 °C until used.

### DNA extraction

Total DNA was extracted from samples using high pure viral nucleic acid kit (Roche) according to the manufacturer’s instructions and eluted in a final volume of 50 μL. Extracted DNA was stored at -80 °C until further use.

### Real-time qPCR assay

Real-time qPCR assay was performed with SYBR® Select Master Mix on Aglient Technologies Stratagene Mx3005P thermocycler (Life technologies) as previously described [3, 23].

### ORFV RPA- LFD assay

Sequences of primers and probe were previously described [23] and designed using parameters according to the TwistDx nfo RPA instruction manual. The reverse primer RPA1R with a 5'-biotine label. The probe consists of an upstream stretch (30 nt) carrying a 5’-FAM antigenic label, which is connected via a THF spacer to an adjacent downstream oligonucleotide (15 nt) carrying a C3-spacer (polymerase extension blocking group) at its 3’ end. All oligonucleotides in this study were synthesized by Sangon Biotech (Shanghai, China).

**Fig. 1:**
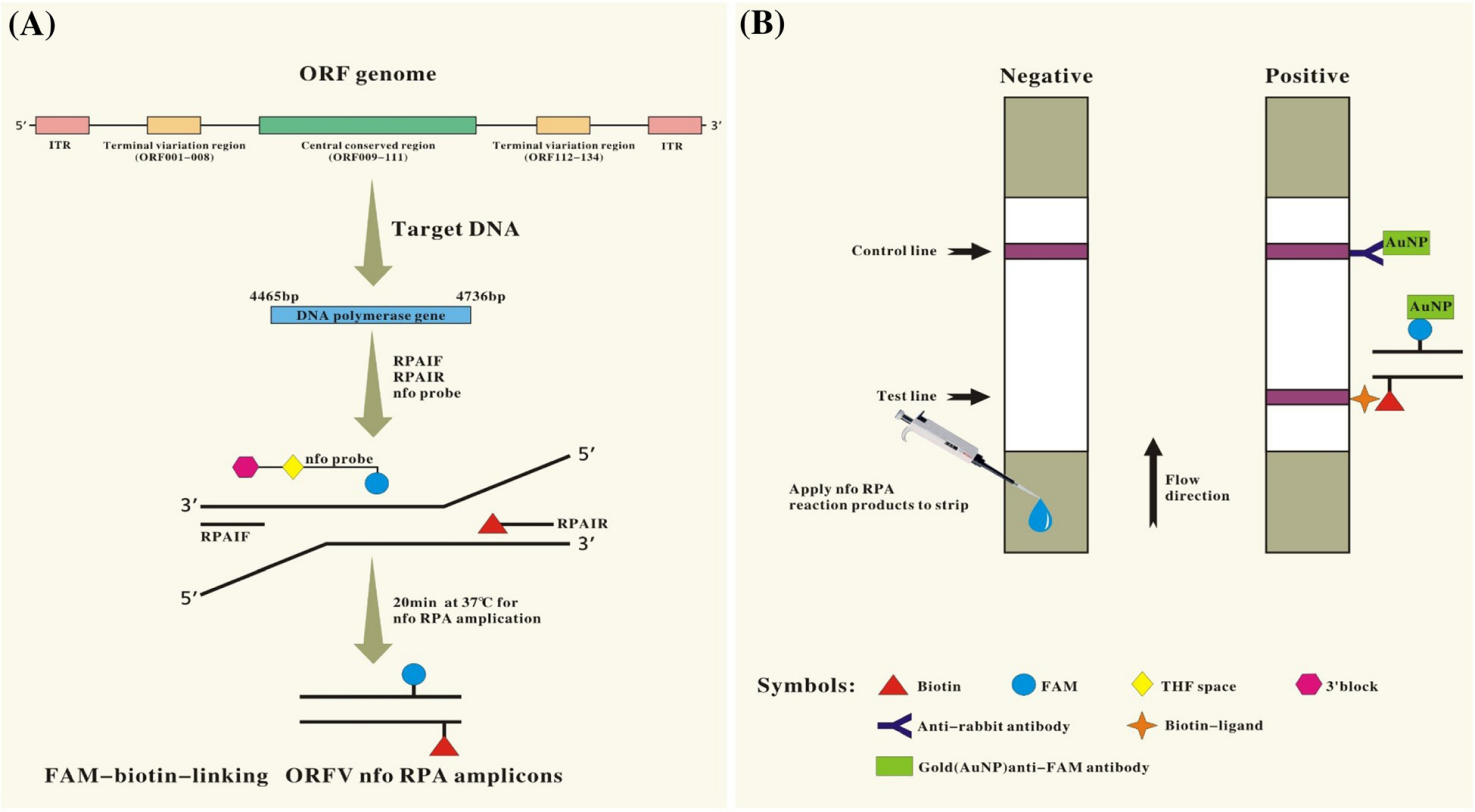
Schematic representation of ORFV RPA-LFD assay principle for the detection of ORFV. **a** Amplifying FAM-biotin-linking ORFV nfo RPA amplicons, in the presence of target template (nt position 4465 bp–4736 bp on ORFV DNA polymerase gene), recombinase nfo endonuclease driven primers (RPA1F/Biotin labeled RPA1R) and FAM labeled probes produced FAM-biotin-linking ORFV nfo RPA. **b** Detecting the RPA amplicons by LFD assay. The amplicons are mixed with the appropriate buffer. Dipping the mixture on LFD strips (Milenia Biotec, Giessen, Germany), the RPA amplicons travel in a buffer stream to be trapped at the test line by biotin-ligands, resulting in an appearance of red-pink color indicative of a positive result. Non-captured gold particles move through the test line to be fixed at the control line by anti-rabbit antibodies, and then produce color serving as a flow control for the strip. In the absence of ORFV target amplicons, color will appear at a control line only

Exo RPA reactions were performed in a 50 μL volume with the TwistAmp nfo kit (TwistDx, UK) contained 420 nM nfo RPA primers, 30 nM RPA probes and 1x rehydration buffer. The reaction were incubated for a typical 20 min at optimized temperature (37 °C), if not indicated otherwise, in a water bath (Fig. 1a).

To detect the RPA-generated, labelled amplicon by LFD, Hybridetect 2 T (Milenia Biotec GmbH, Germany) dipsticks were used. One μL of the RPA product was diluted in 98 μL of the assay buffer (Tris-buffered saline). The LFD strip were directly inserted into the mixture and incubated at an upright position for 2 min. The RPA- generated, labelled product is visualized with gold nanoparticles conjugated with polyclonal anticarboxyfluorescein antibodies (rabbit) contained on the lateral flow strip at the application pad. The gold nanoparticle/amplicon–conjugate can then bind with the 3’-end to immobilize biotin antibodies on the detection line. A control line with immobilized anti-rabbit antibodies serves as an assay control. The entire LFD assay was done at room temperature (Fig. 1b). If necessary, subsequent analysis on 2 % agarose-gel stained with ethidium bromide was carried out to confirm the specific amplification products.

## Results

### Optimization of the ORFV RPA-LFD conditions

**Fig. 2:**
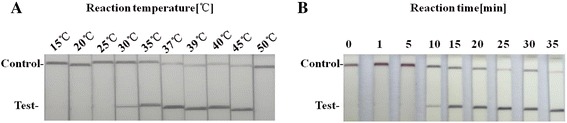
Determination of reaction time and temperature for the ORFV RPA-LFD assay. The assay works effectively in a broad range of constant reaction temperatures (**a**). After 10 min of isothermal amplification reaction, the test line is visible on the test strip. Including the incubation of 5 min the whole assay time of the ORFV RPA-LFD assay is less than 20 min (**b**)

To determine the optimal temperature for the RPA reaction, the ability of the ORFV RPA–LFD assay was tested to amplify 80 copies of ORFV DNA as template at a range of temperatures from 15 °C to 50 °C. Initially, we assessed this range of temperature under incubation time of 20 min. As shown in Fig. 2, no amplification products were observed in reactions incubated at <30 and ≥ 50 °C. There were weak test line at 30 °C, and there were no differences in amplification at 35, 37, 39, 40 and 45 °C (Fig. 2a). Thus, 37 °C was selected arbitrarily as the ORFV RPA-LFD assay standard temperature. We next tested the performance of the ORFV RPA-LFD assay at 37 °C incubated for 1, 5, 10, 20, 25, 30 and 35 min. As shown in Fig. 1b, no amplified products were observed in reactions incubated for less than 5 min and weak amplified product observed for 10 min. When incubation time were increased from 15 to 30 min, the assay performance was improved and there were no differences in amplification in reactions incubated between 15 to 30 min, thus 20 min was selected arbitrarily as standard incubation time of the ORFV RPA-LFD assay in this study.

### Detection limit of the ORFV RPA-LFD assay

**Fig. 3:**
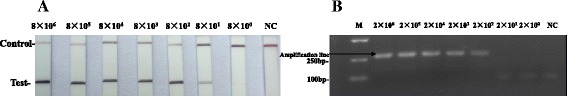
Reaction sensitivity of the ORFV RPA-LFD assay. A serial dilution of the ORFV DNA standard plasmids. NC represent negative control. **a** In the lateral flow format the sensitivity was 80 copies of the ORFV DNA standard plasmids. **b** Positive RPA reaction products (273 bp) can be detect on a stained agarose gel (2 %)

The detection limit of the ORFV RPA-LFD assay was determined using a dilution series of the DNA plasmid standards pOrfv/RP1 (corresponding to 10^1^ to 10^6^ genome copies/reaction) (Fig. 3a) as previously described [23]. Additionally, the amplified products in the ORFV RPA-LFD reaction was also tested by subsequent visualization with agarose gel electrophoresis (Fig. 3b). The results showed that the ORFV RPA-LFD gave clear positive signal at 80 copies/reaction while the agarose gel electrophoresis gave a clear band at 200 copies/reaction. This result indicated the lateral flow dipsticks-based detection has a higher sensitivity than the agarose gel-based detection.

### Specificity of the ORFV RPA-LFD assay

**Fig. 4:**
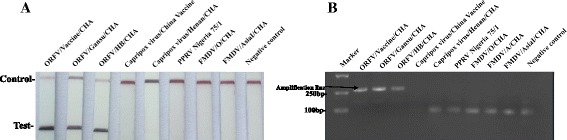
Specificity test results of RPA using total DNA extracted from ORFV and other virus. Positive RPA reaction products can be detect in the lateral flow format (**a**) Positive RPA reaction products (273 bp) can be detect on a stained agarose gel (2 %) (**b**)

In testing specificity of the ORFV RPA-LFD assay at the optimal conditions described above, the reactions were performed using DNA or RNA from ORFV other important viruses of small ruminants which cause similar clinical signs. As clearly showed in Fig, no cross-reactions were observed in the viruses examined. With all other viruses, red-purple color line was only observed at the control line on the LFD strips (Fig. 4a), which was confirmed by the agarose gel electrophoresis (Fig. 4b). The result indicated that ORFV RPA-LFD assay were specific for detection of ORFV.

### Performance of the ORFV RPA-LFD assay on samples

**Fig. 5:**
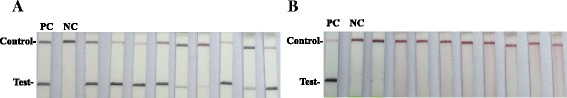
Evaluation of the ORFV RPA-LFD assay for detection of ORFV with qPCR confirmed ORFV-positives and negatives. RPA-nfo reaction was performed as described in the Materials and Methods. **a** nine positive and (**b**) nine negative samples were evaluated along with positive (PC) and negative (NC) controls

Initially, the feasibility of the ORFV RPA-LFD assay for diagnosis was tested validated with known positive samples (*n* = 9) and negative samples (*n* = 9) which was confirmed by ORFV qPCR assay. As showed in Fig. 5, the ORFV RPA-LFD assay was able to be 100 % correct to identify all samples. Its feasibility was then further validated with samples (*n* = 53) collected from goats with suspected orfv infection, eight nasal swabs collected from experimentally infected sheep and five samples obtained from healthy goats. Of 53 samples collected from suspected goats of the Orf, 17 samples were found to be positive by ORFV RPA-LFD assay, the results were identical to the results of real-time ORFV qPCR assay (CT value ranging from 15.3 to 32.8). Furthermore, all of the nasal swabs (*n* = 8) collected from experimentally infected sheep and ORFV-spiked tissues lysates (*n* = 24) were positive while samples (*n* = 5) obtained from healthy goats were negative by ORFV RPA-LFD assay. Based on a total of 90 samples examined, the sensitivity and the specificity of ORFV RPA-LFD assay for identification of ORFV was 100 and 100 % respectively when compared to real-time ORFV qPCR (Additional file 1: Table S1).

## Discussion

In this study, we development a RPA in combination with LFD described for rapid visual detection of ORFV, which is minimally instrumented and has the potential to used as a point of care diagnostic tool. The results have showed that the developed ORFV RPA-LFD assay has an enough wide range of temperatures which the assay can tolerate because there were no differences in amplification with the range from 30 to 45 °C. It is an important feature for a point of care assay which may be used under uncontrolled temperature environment in the field. More importantly, this result can be read out as early as 20 min (15 min reaction time plus 5 min on LFD).

Recently, another isothermal amplification method for detection of ORFV was developed based on LAMP technology [6]. In contrast to the PPA, LAMP requires a larger set of six primers, a higher temperature (62 °C) and a longer run time. The sensitivity of RPA assay usually presents equal to LAMP assay area, but its specificity is higher that the LAMP assay. The developed ORFV RPA-LFD assay results demonstrated an optimal specificity. Testing results of the ORFV and other related virus showed an analytical specificity of 100 % compared to the qPCR assay and no cross-reaction with non-ORFV viruses. The result has also showed that the assay has high sensitivity for detection of ORFV (80 copies/reaction), which is higher than 200 copies/reaction detected by a fluorescent probe-based RPA assay [23]. Nonetheless, this level of sensitivity should be sufficient to detect ORFV present in samples collected from infected animals. Further assessment of the assay’s performance with clinical samples has demonstrated satisfactory performance in term of specificity and sensitivity compared to the qPCR assay. Based on results in this study, the ORFV RPA-LFD assay has great potential as a point-of-care molecular diagnostic assay for detection of ORFV because of its simplicity, minimal dependency of any specified instrument (portability) and short reaction times. In the developed ORFV RPA-LFD assay, simple instrumentation is needed for both RPA reaction itself and the analysis on the LFD, resulting in overall lower diagnostic costs, which is ideal for onsite testing. A visible band on the LFD strip gives a clear positive/negative answer, which can be easily identified by the naked eyes without any training. In the context of the ever-expanding epidemic of many viral infection in animals and humans over the past years, there is an urgent need for affordable molecular detection assay with good sensitivity and specificity for viral diagnostic in developing countries, where no such available assays are currently affordable or accessible. Such availability of more affordable assays would also fill this gap because of decentralization of testing in developing countries.

## Conclusions

The developed ORFV RPA-LFD assay is a sensitive and specific method for rapid visual detection of ORFV, and it has great potential as a point-of-care molecular diagnostic assay. However, the effectiveness of this assay for diagnosis of Orf must be fully evaluated with a larger number of ovine samples before this assay can be considered for routine diagnostic tool.

## Additional file

Additional file 1: Table S1. Comparison of ORFV RPA-LFD assay with qPCR assay on ORFV spiked samples and clinical samples. (DOC 26 kb)

## Abbreviations

LAMP: loop-mediated isothermal amplification
ORFV: Orf virus
qPCR: quantitative PCR
RPA: recombinase polymerase amplification
RPA-LFD: recombinase polymerase amplification in combination with a simpler lateral flow immunoassay strip
