# Supplementary material for: Development of an isothermoal amplification-based assay for rapid visual detection of an Orf virus
Source: Virol J. 2016 Mar 19;13:46. doi: 10.1186/s12985-016-0502-x (PMC4799565; doi:10.1186/s12985-016-0502-x)
Supplement: Additional file 1: Table S1. — Comparison of ORFV RPA-LFD assay with qPCR assay on ORFV spiked samples and clinical samples. (DOC 26 kb) [file 12985_2016_502_MOESM1_ESM.doc]

Table S1. Comparison of ORFV RPA-LFD assay with qPCR assay on ORFV spiked samples and clinical samples*

| qPCR  Positive Negative |
| --- |
| RPA Positive 41 0 41  Negative 0 49 49  41 49 90 |

*Samples include twenty-four ORFV-spiked tissues samples, fifty-three samples collected from goats with suspected orfv infection, eight nasal swabs samples, and five tissues samples from healthy goats. All samples either ORFV or no viral DNAs detected.
